# Effects of flower thrips (Thysanoptera: Thripidae) on nutritional quality of banana (Zingiberales: Musaceae) buds

**DOI:** 10.1371/journal.pone.0202199

**Published:** 2018-08-10

**Authors:** Deyi Yu, Peng Huang, Yong Chen, Yongwen Lin, Komivi Senyo Akutse, Yanyang Lan, Hui Wei

**Affiliations:** 1 Institute of Plant Protection, Fujian Academy of Agricultural Sciences, Fuzhou, China; 2 Fujian Key Laboratory for Monitoring and Integrated Management of Crop Pests, Fuzhou, China; 3 Plant Protection College, Fujian Agriculture and Forestry University, Fuzhou, China; 4 International Centre of Insect Physiology and Ecology, Nairobi, Kenya; 5 Research and Development Centre of Zhangzhou National Agricultural Science and Technology Zone, Zhangzhou, China; Fred Hutchinson Cancer Research Center, UNITED STATES

## Abstract

The abundance of banana flower thrips (*Thrips hawaiiensis* Morgan) in a banana (*Musa acuminata* Colla “Williams” cultivar) plantation was investigated using yellow sticky traps (29.70 cm × 21.00 cm) in 2015. Banana flower thrips occurred throughout the year with monthly variation, and the maximum occurrence was observed in October and November during the bud burst (73.80 ± 6.32 adults/trap) and young fruit (70.06 ± 5.69 adults/trap) periods. The damage rates were as follows: interior flowers >3^rd^-layer flowers > 2^nd^-layer flowers > 1^st^-layer flowers > young fruits. This result indicates that thrips migrated to lower bracts, young fruits, and other flower buds as bracts gradually opened. Results also showed that the reducing sugar, vitamin C, protein and ash contents in thrips-damaged flowers were all significantly lower than those in undamaged flowers, while there was no significant difference between damaged and undamaged young banana fruit. Our results indicated that the abundances of banana flower thrips were closely associated with the growing stage of banana. Thrips mainly infested flower buds and caused a reduction in nutrients for the host plant, especially the reducing sugar and vitamin C contents, which reduced the nutritional quality of banana fruits and the quality of flower bud by-products of banana.

## Introduction

Banana (*Musa acuminata*) is the world’s most important fruit crop and one of the top 10 crops by production [1]. It is widely grown in the tropics and subtropics, where it acts as an important dietary component, both raw (as a dessert fruit) and cooked (often as the major source of carbohydrates) [2]. Banana produces a large number of flower buds, which have been developed for edible and pharmaceutical functions due to their abundance of nutrients and medicinal contents [3, 4]. In recent years, banana flower thrips (*Thrips hawaiiensis* Morgan) that damage flower buds have become major pests of banana crops [4–6]. The thrips cause dark-brown and black bumps on the surface of flower buds and young fruit through their rasping-sucking and oviposition behaviors [6]. Theoretically, *T*. *hawaiiensis* not only impacts flower nutritional quality, edibility, and pharmaceutical value but also affects the development and utilization of banana bioproducts. It is necessary to establish reasonable management methods to control banana flower thrips [7].

The strategy for controlling banana flower thrips in the field is the use of chemical insecticides that not only kill targets but also result in “3R” (residue, resistance and resurgence) [8] and phytotoxicity problems [9, 10]. Some researchers tried sticky traps and attraction based on the susceptibility of thrips to particular colors or volatile compounds [11, 12]. However, this approach is more likely to monitor pests’ occurrence than reduce their populations. Using resistant host species is one of the most effective approaches to control pests and improve crop quality, as it reduces the infestation by pests at the source [13, 14]. For using this method, it is necessary to investigate the characteristics of damage caused by target pests to the host plant and the effect of pest feeding on the nutritional quality of the host plant [12, 15]. Thrips intake nutrition for growth, reproduction, and physiological metabolism by piercing the host plant and sucking up protein, carbohydrates, lipids, vitamins, water, inorganic salts, and other nutrients. This process not only changes the nutrient contents of the host plants but also causes compensation, eventually affecting the resistance and nutritional quality of the host plant [16, 17].

A previous study showed that the damage characteristics of banana flower thrips and its preference for “Williams” banana, “red” banana, and “pink” banana flower buds and young fruits[18]. However, the influences of flower thrips on the biology of banana flowers and young fruits are still unknown. Here, the abundance of banana flower thrips was observed in a “Williams” banana plantation in Tianbao Town, Zhangzhou City. Before the buds were cut off, six nutritional indicators, including protein, reducing sugar (easily absorbed by insects), crude fat, ash (consisting of inorganic salts and oxides), vitamin C (improves plant resistance), and water were compared among different flower layers and young fruits. The effects of banana flower thrips on the nutritional quality of banana flower buds were clarified to provide technical support for the management of thrips and banana flower buds.

## Materials and methods

### Crop culture

The *M*. *acuminata* “Williams” cultivar was selected for this study. Banana crops were planted in the field in an area of approximately 2.3 hm^2^ located in Tianbao Town, Zhangzhou City (23°34′53″ N, 117°32′14″ E). Banana plants were grown in a deep-furrow border-check system (4.0–4.5 m in border-check width, 0.5–0.7 m in furrow width, 0.4–0.6 m in depth). The spacing of individual plants was 2.0–2.5 m, and the height of plants varied from 2.0–2.5 m.

### Classification of developmental stages

**Fig 1:**
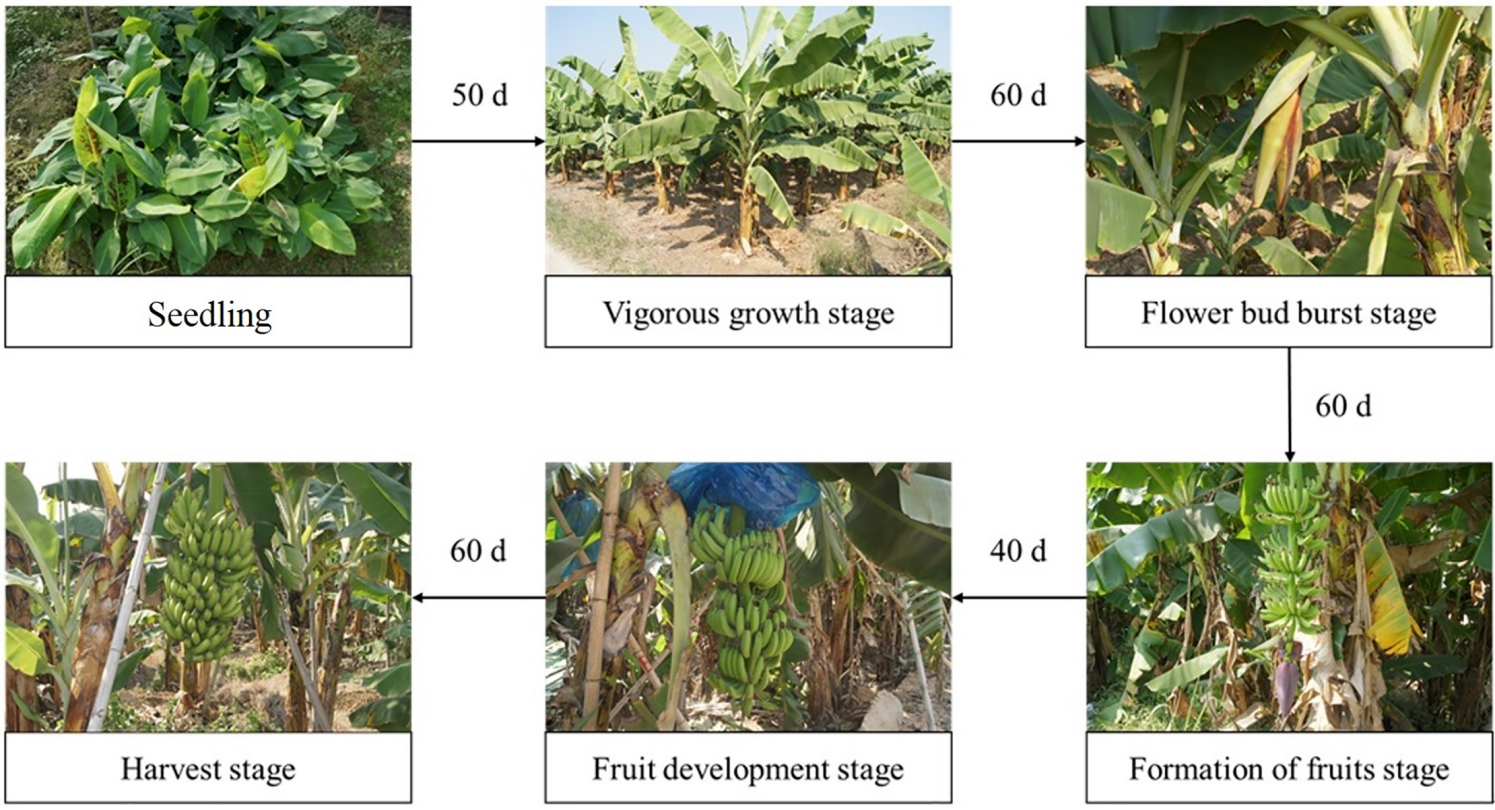
Pictures of the different developmental stages of banana.

The developmental stages of banana in Tianbao Town were classified as seedling stage (fifty days from planting in the field), vigorous growth stage (sixty days), flower bud burst stage (sixty days), formation of fruits stage (forty days), fruit development stage (sixty days) and harvest stage (thirty days) (Fig 1).

### Sampling method

Twenty 40-day-old seedling plants were selected for tissue culture, randomly sampled in a "Z" pattern and labeled for observation at 10-day intervals. The damage rate of plants was recorded in all the plant tissues (stems, leaves, flower buds, and fruits) during the whole growth period.

### Population dynamics of thrips

The twenty selected banana plants mentioned above were investigated, and the population dynamics of banana flower thrips were analyzed as described by [19]. Bamboo poles (1.7 m high) used to hang yellow sticky traps (29.7 cm × 21 cm) were stuck into the earth, 50 cm away from the sampled plants. The sticky traps were 1.5 m above the ground. The number of thrips on each sticky trap was recorded every 10 days using a 40× magnifier. The sticky traps were replaced every 10 days. This experiment was repeated 3 times.

### Preference of thrips for different tissues

Ten thirty-day-old thrips-free inflorescences (3 layers of young fruit) were covered with insect net (50 mesh) bags, and one hundred banana flower thrips were then transferred into each bag for twenty days. One fruit and flower bud from each layer of inflorescence on the selected banana trees were marked and collected in black transparent bags. All samples were transported to the lab immediately to assess the damage rate. All the dark spots (S1 Fig) on the surface of each sample (young fruit and flower bud) were recorded under a 20X binocular microscope (SZ760, Chongqing Optec Instrument Co., Ltd., Chongqing, China). The severity of damage to fruits and flowers was divided into four levels: L0, undamaged; L1, < 10 spots; L2, < 30 spots; and L3, > 30 spots. Thrips-free inflorescences were used as a control, and this experiment was repeated five times.

### Nutritional quality of flower buds and young fruit

The protein, reducing sugar, crude fat, ash, vitamin C, and water contents in each sample were measured in the same layers for samples with different damage rates. The L0 samples were regarded as the control (CK) and the experiment was repeat for 5 times. The protein content was determined with the method described by Kjeldah [20], reducing sugar content was determined using direct titration [21], crude fat content was extracted using Soxhlet extraction [22], ash content was determined with the method described by Dinuzzo et al. [23], vitamin C content was determined using 2,6-dichloroindophenol titration [24], and water content was determined using direct drying [25].

### Statistical analysis

The mean values of preference damage rate and nutritional quality were compared by Tukey’s HSD test (α = 0.05) following analysis of variance using SPSS 20.0 (Microsoft, USA).

## Results

### Abundance of banana flower thrips

**Fig 2:**
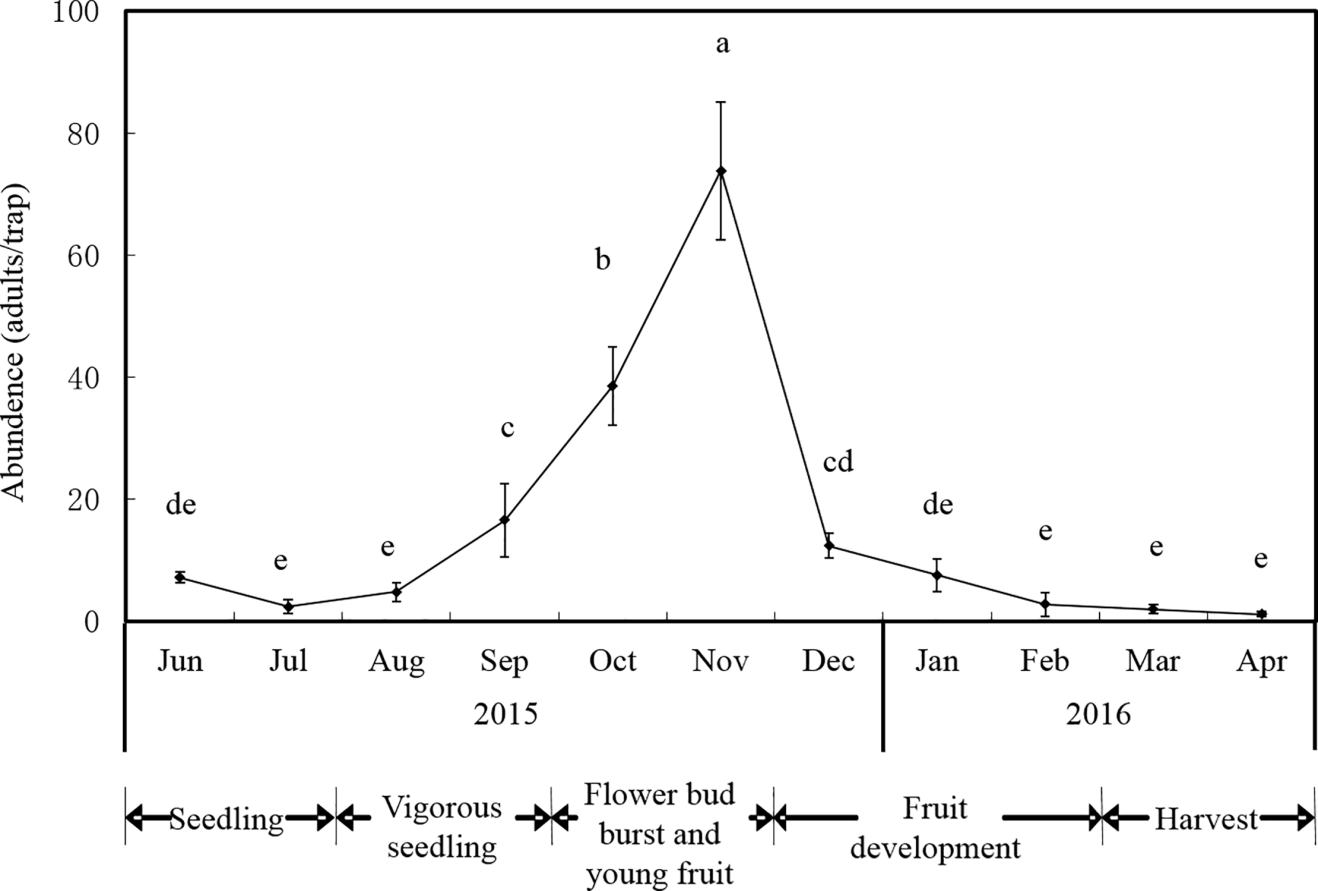
Seasonal abundance of *Thrips hawaiiensis* caught on sticky yellow traps in Zhangzhou from 2015 to 2016. Different latters above bars indicate significant differences at P < 0.05.

Thrips appeared sporadically in young leaves during the seedling period, while stems, leaves, and other tissues did not show any sign of damage during this growth stage. Thrips started rasping-sucking and oviposition behavior on the flower buds from the bud burst to young fruit stage. This damage became gradually worse before the flower clusters were cut off. Thrips invaded tissues before buds opened and then migrated to the lower bracts, young fruits, and other flower buds as the bracts gradually opened. The abundance of thrips decreased rapidly when plants were covered with fruit bags during the fruit developmental stage. No additional damage was observed at the end of the harvest. Thrips occurred throughout the year, and the population abundance fluctuated widely from July to December (Fig 2). The abundance of thrips in the bud burst and young fruit stages, which occur in October-November, was higher than that in the other stages (*F*_2,5_ = 121.78, *P* = 0.0001). The abundance observed on seedlings and during the vigorous growth stage (June-August) was significantly lower than that of the other stages (*F*_2,5_ = 28.37, *P* = 0.017). The abundance rapidly increased to 73.80 adults/trap in October-November (flower bud burst and young fruit stages) and was significantly higher than in any other month (*F*_2,5_ = 18.52, *P* = 0.012). The abundance sharply declined (0.80–7.60 adults/trap) in December (fruit development stage) and then maintained a smooth downward trend in the following months (January-May).

### Damage rates in different tissues

**Fig 3:**
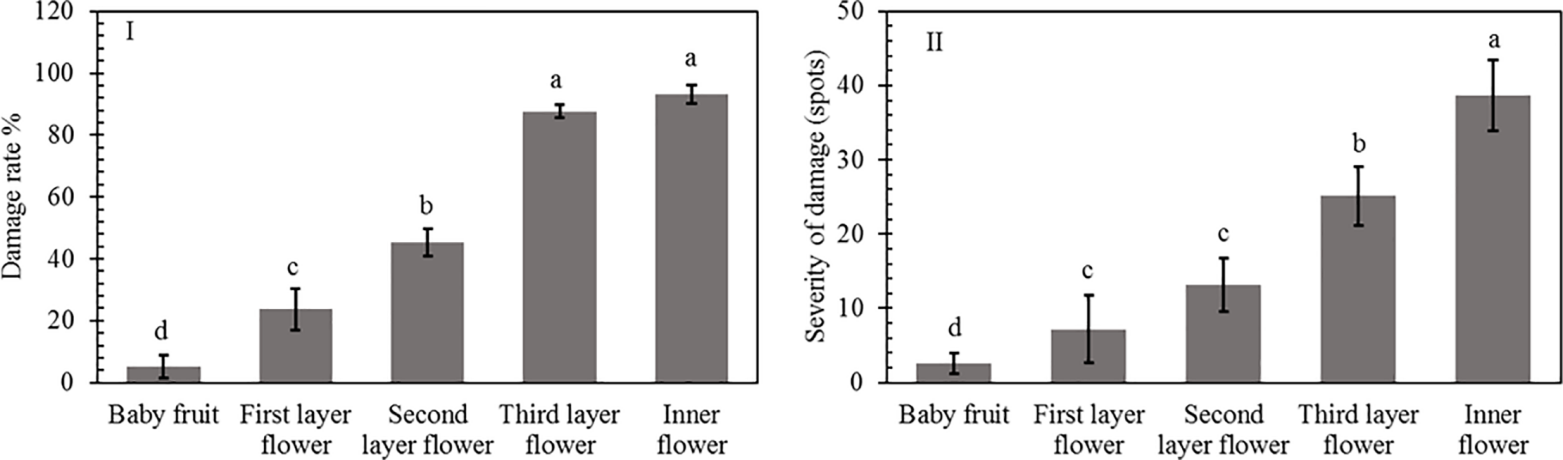
Damage rates in different tissues attacked by banana flower thrips. I, damage rate of banana flowers and young fruits caused by thrips; II, severity of damage to banana flowers and young fruits caused by thrips. Different letters above bars indicate significant differences at P < 0.05.

The damage rates were ranked as follows: interior flowers > 3^rd^-layer flowers > 2^nd^-layer flowers > 1^st^-layer flowers > young fruits (Fig 3-I). Damage occurred significantly more often in the inner flowers (93.12%) and third-layer flowers (87.56%) than in the other 3 tissues (*F*_4,4_ = 44.75, *P* = 0.0001). A significant difference in damage rate was also found between the first-layer flowers (23.69%) and 2^nd^-layer flowers (45.13%) (*F*_4,4_ = 13.57, *P* = 0.024). The lowest damage rate (5.17%) was observed in the young fruit. There was no significant difference in damage rate between the 3^rd^-layer flowers and interior flowers (*F*_4,4_ = 0.89, *P* = 0.78).

For the severity of damage, we also found the following rank: interior flowers > 3^rd^-layer flowers > 2^nd^-layer flowers > 1^st^-layer flowers > young fruits (Fig 3-II). The severity of damage to interior flowers (38.69 spots) was significantly higher than that to the other tissues (*F*_4,4_ = 54.76, *P* = 0.003), while the severity of damage to young fruits (2.58%) was significantly lower than that to the other tissues (*F*_4,4_ = 33.89, *P* = 0.011).

### Effects of thrips on nutritional quality

As shown in Table 1, the protein, reducing sugar, crude fat and vitamin C contents in thrips-damaged flower were 14.25%, 6.00%, 6.20% and 17.20%, all significantly lower than in undamaged flower (control)(*F*_1,4_ = 22.17, *P* = 0.010; *F*_1,4_ = 10.41, *P* = 0.036; *F*_1,4_ = 17.99, *P* = 0.021; *F*_1,4_ = 93.41, *P* = 0.001). Thereafter, the protein content of the 2nd flower layer rebounded due to host plant compensation, but this did not counter the loss caused by thrips damage. Hence, the protein content of the 1st flower layer showed a significant decrease and then recovered to the level of the 3rd flower layer (*F*_1,4_ = 18.65, *P* = 0.018). Similar effects of plant compensation were observed for vitamin C in the 3rd flower layer. The vitamin C content even significantly higher than that of the control (*F*_1,4_ = 38.41, *P* = 0.002). A continuous, significant decline in vitamin C was observed in the 1st and 2nd flower layers (*F*_1,4_ = 187.35, *P* = 0.0001; *F*_1,4_ = 105.33, *P* = 0.0001). There was no significant difference in water content among the different flower layers, although it decreased in the range of 90.30 to 91.61 g/100 g (*F*_4,16_ = 1.25, *P* > 0.05).

**Table 1:** Effects of banana flower thrips on the nutritional quality of different flower layers.

Nutritional content	1^st^-layer flowers	2^nd^-layer flowers	3^rd^-layer flowers	Interior flowers	Average	Control (CK)
Protein content (g/100 g)	13.10±0.02d	15.00±0.04c	13.30±0.09d	15.60±0.17b	14.25±0.07e	17.33±0.48a
Reducing sugar content (g/100 g)	4.00±0.07d	6.30±0.13c	6.80±0.14b	6.90±0.05b	6.00±0.05e	7.24±0.30a
Crude fat content (g/100 g)	6.00±0.13bc	6.20±0.07b	6.30±0.12b	6.30±0.03b	6.20±0.07b	7.50±0.37a
Ash content (inorganic salt) (g/100 g)	12.20±0.30d	12.30±0.20cd	12.60±0.40c	14.10±0.10a	12.80±0.20b	13.10±0.35b
Vitamin C content (mg/100 g)	9.60±0.27e	13.80±0.18d	18.80±0.27a	16.40±0.18c	14.65±0.07f	17.20±0.60b
Water content (g/100 g)	90.32±0.47a	90.57±1.50a	91.04±0.14a	91.61±0.48a	90.89±0.58a	90.84±0.43a

Note: Means ± SE followed by different alphabets represent significant differences at *P* < 0.05.

The effect of thrip damage on the nutritional quality of young fruit was showed in Table 2. The content of reducing sugar and vitamin C in thrips-damaged young fruit were 4.91% and 9.60, both significantly lower than in undamaged one (control) (*F*_1,4_ = 9.82, *P* = 0.030; *F*_1,4_ = 53.87, *P* = 0.0001). The changes in protein, crude fat, ash, and water contents were not significant (*P >* 0.05). Protein and water contents decreased slightly. Conversely, crude fat and ash contents increased slightly and fluctuated in the range of 0.90% to 1.64% (*F*_1,4_ = 1.98, *P* = 0.121; *F*_1,4_ = 1.13, *P* = 0.213).

**Table 2:** Effects of banana flower thrips on the nutritional quality of young fruits.

Nutritional content	Damaged	Undamaged (control)	*P*
Protein content (g/100 g)	12.80 ± 0.02a	13.01 ± 0.03a	0.1508
Reducing sugar content (g/100 g)	4.91 ± 0.02b	5.31 ± 0.05a	0.0308
Crude fat content (g/100 g)	6.69 ± 0.01a	6.63 ± 0.03a	0.1004
Ash content (inorganic salt) (g/100 g)	19.18 ± 0.03a	18.89 ± 0.02a	0.3203
Vitamin C content (mg/100 g)	9.60 ± 0.02b	12.51 ± 0.04a	0.0001
Water content (g/100 g)	92.88 ± 0.28a	94.12 ± 0.21a	0.0740

Note: Means ± SE followed by different alphabets represent significant differences at *P* < 0.05.

## Discussion

Understanding the host preference of target pests, their abundance and their influence on nutritional quality are prerequisites for the application of resistant cultivars, which are one of the most effective strategies of pest control [26, 27]. Thrips damaged flower buds by rasping-sucking and ovipositing throughout the year. The order of damage rates was as follows: interior flowers > 3^rd^-layer flowers > 2^nd^-layer flowers > 1^st^-layer flowers > young fruit. Thrips abundance was closely related to banana growth stage and varied widely each month. The highest abundance was observed during the bud burst and young fruit stages (October-November). This finding indicated that flower buds are a defining characteristic of determination materials used for breeding resistant cultivars.

Plants have evolved a series of complicated defense mechanisms against insect attacks. Plants supply nutrients, including protein, carbohydrates, lipids, vitamins, water, and inorganic salts, that are necessary to maintain insect life, but they are also induced to produce a series of physiological and biochemical reactions to compensate for the damage caused by insects accessing these resources [28, 29]. Many researchers have focused on the interspecific relationship between plants and herbivores, such as the photophysiological responses of alfalfa *Medicago sativa* to thrips damage (*Odontothrips*) [30], the effects of rasping-sucking by thrips (*Gynaikothrips uzeli*) on the chemical constitution of surface wax and amino acid content in *Ficus benjamina* [31], the relationship between soybean aphid (*Aphis glycines*) damage and physiological indexes of soybean (*Glycine max*) leaves [32], and the response of secondary metabolism of plane tree (*Platanus acerifolia*) leaves to tree bug (*Corythucha ciliata*) damage [33]. However, there are few studies that identify the resistance and nutritional changes of banana attacked by banana flower thrips. In our study, the nutritional contents (protein, reducing sugar, crude fat, ash, vitamin C, and water) were compared between damaged and undamaged flowers and young fruits. The results showed that all indexes fluctuated with damage level, except for water content. Plant compensation appeared in the 2nd and 3rd flower layers with higher protein and vitamin C contents but did not equal the loss caused by thrips damage. Overall, the nutritional values decreased with increasing damage, and the same results were found in previous studies [31–34]. There were significant decreases in reducing sugar and vitamin C contents in young fruit but not in the other nutritional contents. These results not only confirmed the complicated and subtle interspecific relationship between banana and thrips but also provided evidence to quantitatively evaluate the benefits and costs to buds that have been attacked by thrips.

Our evaluation of the nutritional quality of buds also showed the benefits and costs to physiological indexes caused by thrips attacks. This could also explain how plants and herbivorous insects coevolved, but it is not clear how plant metabolism, such as ultramicrostructural tissues, secondary substances (phenolic compounds, terpenoids), and enzyme activities, could change. Therefore, the effects of thrips attacks on morphological structures, secondary substances, and enzyme activities should be included in future studies to clarify the function of flower buds and establish suitable management methods for thrips.

In conclusion, the degree of damage to banana plants depended on *T*. *hawaiiensis* abundance. Thrips mainly invaded flower buds and caused poor nutritional quality of fruits and their bud by-products, particularly in reducing sugar and vitamin C contents.

## Supporting information

S1 Fig Damage symptoms of banana flower thrips against banana flowers and baby fruit. The tiny black points in the red circles were damage symptoms of banana flower thrips.
(DOCX)
