# Supplementary material for: Effects of flower thrips (Thysanoptera: Thripidae) on nutritional quality of banana (Zingiberales: Musaceae) buds
Source: PLoS One. 2018 Aug 10;13(8):e0202199. doi: 10.1371/journal.pone.0202199 (PMC6086469; doi:10.1371/journal.pone.0202199)
Supplement: S1 Fig — The tiny black points in the red circles were damage symptoms of banana flower thrips. (DOCX) [file pone.0202199.s001.docx]

**Supporting information**


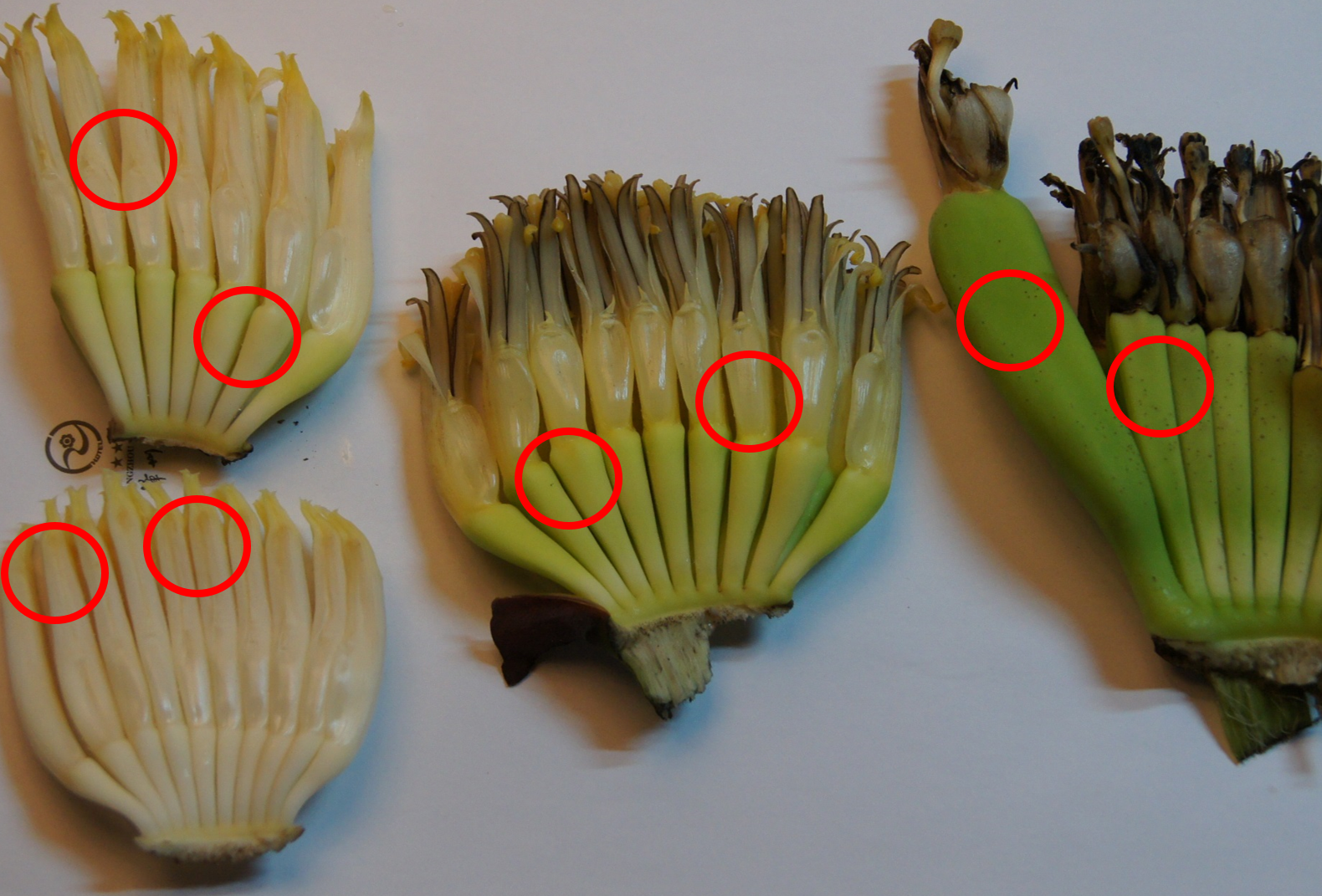


**S1 Fig. Damage symptoms of banana flower thrips against banana flowers and baby fruit. The tiny black points in the red circles were damage symptoms of banana flower thrips**
